# LeanNet: An Efficient Convolutional Neural Network for Digital Number Recognition in Industrial Products

**DOI:** 10.3390/s21113620

**Published:** 2021-05-22

**Authors:** Na Qin, Longkai Liu, Deqing Huang, Bi Wu, Zonghong Zhang

**Affiliations:** The Institute of Systems Science and Technology, Southwest Jiaotong University, Chengdu 610031, China; qinna@swjtu.cn (N.Q.); m13653974033@163.com (L.L.); auggie1996@my.swjtu.edu.cn (B.W.); zzh451045814@163.com (Z.Z.)

**Keywords:** convolutional neural network, image classification, network pruning, MobileNet, SqueezeNet

## Abstract

The remarkable success of convolutional neural networks (CNNs) in computer vision tasks is shown in large-scale datasets and high-performance computing platforms. However, it is infeasible to deploy large CNNs on resource constrained platforms, such as embedded devices, on account of the huge overhead. To recognize the label numbers of industrial black material product and deploy deep CNNs in real-world applications, this research uses an efficient method to simultaneously (a) reduce the network model size and (b) lower the amount of calculation without compromising accuracy. More specifically, the method is implemented by pruning channels and corresponding filters that are identified as having a trivial effect on the output accuracy. In this paper, we prune VGG-16 to obtain a compact network called LeanNet, which gives a 25× reduction in model size and a 4.5× reduction in float point operations (FLOPs), while the accuracy on our dataset is close to the original accuracy by retraining the network. Besides, we also find that LeanNet could achieve better performance on reductions in model size and computation compared to some lightweight networks like MobileNet and SqueezeNet, which are widely used in engineering applications. This research has good application value in the field of industrial production.

## 1. Introduction

In recent years, we have witnessed a rapid development of CNNs in various fields such as image encryption [1], object detection [2], semantic segmentation [3], protein function prediction [4] and many others. CNNs can achieve a desirable performance thanks to large-scale datasets, GPUs with efficient parallel computing power and their own strong fitting capacity. Though large CNNs are very powerful, they consume considerable storage, computational resources and energy resources. For instance, the VGG-16 [5] has 138.34 million parameters, takes up more than 500 MB storage space, and requires 15.61 billion FLOPs to classify a single image with a resolution of 224 × 224. This makes it difficult to deploy CNNs on some devices used in practical engineering projects, such as embedded devices and mobile devices.

In the face of the actual demand for the application of CNNs in industry, the issue of difficult deployment of CNNs should be solved urgently. Due to the limited resources of embedded and mobile devices, the following considerations must be taken into account while deploying CNNs: *(1) Model size*: The larger CNNs, have stronger representation power and contain more parameters. It is because of a large number of parameters that the neural network can theoretically approximate any complex function with any accuracy. However, the demand of deploying a 500 MB VGG-16 model on embedded devices is impractical; *(2) Memory footprint*: During the inferencing process, the model generates a large number of calculations, which usually takes up more memory than the model itself. For example, with the VGG-16 batch size set to one, the forward propagation process will take up about 900 MB, which is unaffordable for some resource-constrained platforms; *(3) Inference speed*: Because of the complexity of the model structure and too much computation, it often takes several seconds to inference high-resolution images, but the harm caused by high latency in practical applications such as autopilot is hard to accept.

To reduce the huge overhead of deploying CNN models, many studies are performed to compress the scale of CNNs and speed up the inferencing process. These include network pruning, weight quantization, low-rank approximation and knowledge of distillation. The above methods start from the structure of the model and seek an optimal network structure or replace the floating-point number with a fixed-point number. Because of a large design space for neural networks, another direction is to design lighter-weight network structures directly. For example, MobileNet [6] is based on a pipelined architecture that repeatedly uses depthwise separable convolutions to structure lightweight deep neural networks (DNNs). SqueezeNet [7] uses a new block named *FIRE module* to get a small CNN architecture.

**Figure 1:**
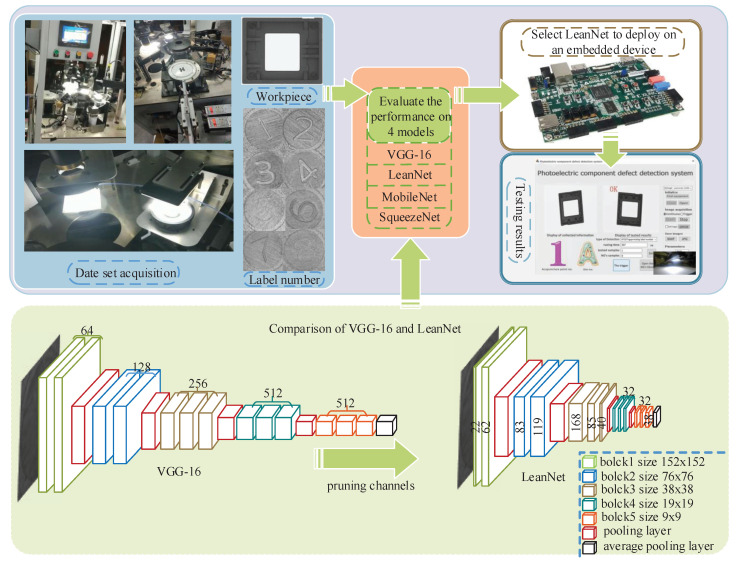
Flowchart of the whole project. The above side shows the process from acquiring the data set to evaluate the model and finally to deploy the model, the below side shows and compares the structure of VGG-16 and LeanNet in detail. The label number part of the flowchart is a collection of label parts on some industrial electronic equipment parts, from 1 to 8.

In this study, to check whether the mold of each workpiece works normally and meets the engineering needs, the label number of each workpiece needs to be identified with high precision and high speed. To meet the requirement of high accuracy, VGG-16 is adopted for label number identification, and the channel pruning method is used to prune VGG-16 to meet the deployment requirement. We refer to the pruned VGG-16 as LeanNet. Figure 1 shows the whole process of this project. Experiments on our dataset show that it can achieve a 25× reduction in model-size and 4.5× reduction in FLOPs while regaining close to the original precision by retraining the networks.

We then compare LeanNet with other state-of-the-art models: MobileNet and SqueezeNet. Experimental results show that LeanNet is significantly better than MobileNet and SqueezeNet. MobileNet is about as accurate as LeanNet but the model is five times larger. SqueezeNet’s model is about the same size as LeanNet but has a 3% lower accuracy on the test set. Overall, the main contributions of the paper are summarized as follows:

- The proposed LeanNet can effectively reduce the parameters and computational complexity, and the accuracy on our dataset is close to the original accuracy by retraining the network, so it can be deployed on some devices with limited computing resources.
- Experimental results show that LeanNet achieves better performance on reductions in model size and computation compared to some lightweight networks like MobileNet and SqueezeNet.

The rest of this paper is organized as follows: Related work is discussed in Section 2. We will introduce the details of LeanNet in Section 3. The experiments are presented to compare LeanNet with other lightweight networks in Section 4. In Section 5, we discuss the results. Finally, the work is concluded in Section 6.

## 2. Related Work

**Low-rank approximation**. To reduce the computational cost of DNNs, a low-rank approximation method is proposed, which represents the weight matrix as a low-rank product of two smaller matrices [8,9,10]. These works have achieved a good effect of acceleration and compression on DNNs with 1% accuracy drop. However, there are some problems when this method is applied to compress the structure of DNNs. For example, the low-rank approximation can only be applied to each layer of the network, and during the fine-tuning, the structure of each layer of the network is fixed, so the time cost of decomposing and fine-tuning the model is expensive. With the linear growth of hyperparameters of each layer in the low-rank approximation method, the search space for the optimal structure will also grow linearly for very deep DNNs [11,12].

**Network pruning**. Pruning is a very efficient and intuitive approach to make neural networks more compact and faster. In the early, [13] introduces *Optimal Brain Damage*, it removes neurons those make little or no contribution to the output of a trained network. Later, Hassibi and Stork propose *Optimal Brain Surgeon* to remove trivial weights determined by the second-order derivative information [14]. However, this method is costly for today`s DNNs. Han et al. [15,16] reduce redundant parameters to get an irregular sparse matrix by a three-stage pipeline that contains pruning, quantization, Huffman encoding. Since parameters are mainly concentrated in the fully connected layer, the author obtains 3× layer-wise speedup. However, in the convolutional layer, no practical speedups are observed, besides, this method can only achieve acceleration with specific sparse matrix operation libraries or hardware, thus it seems to be less practical in real-world applications.

Recently, some researchers have overcome this limitation. Ref. [17] presents an approach that utilizes neuron-level sparsity during network training, hence some neurons could be removed to get a small network. Ref. [18] proposes the pruning filters method, they prune filters from CNNs that are identified as having a small effect on the output accuracy, which yields more compact networks with comparable precision. Ref. [19] proposes a *Structured Sparsity Learning* method to regularize the structures (i.e., filters, channels, filter shapes, and layer depth) of DNNs. Another simple and effective method of channel pruning was proposed in [20]. They use a trainable parameter γ from batch normalization (BN) layers as scaling factors and regularize them, and then train the network and scaling factors at the same time. Finally, they prune channels with small scaling factors and fine-tune the pruned network to obtain an efficient network. In this research, this method is leveraged to prune VGG-16. The aforementioned methods are known as structural pruning, they usually do not need to use specific hardware or software to achieve inference speedup and memory footprint saving.

**Weight quantization**: Weight quantization is the process of converting a continuous weight range into several discrete points so that weights can be represented by fewer bits. For instance, if original weights can be divided into eight groups by clustering methods, only three bits are required to represent indexes of these weights, and the storage space can be greatly reduced by storing index values and corresponding weights. Han et al. [16] leverage the weight quantization method to compress the model three times. Reference [21] proposes a method to quantify parameters (represent all parameters with a small number of parameters) and to estimate the output of convolutional layers and fully connected layers by inner product operation. Weight quantization can usually be used with other network compression and acceleration methods, Ref. [22] combines network pruning and weight quantization in a single learning framework that performs pruning and quantization jointly, and in parallel with fine-tuning.

The extreme way of weight quantization is network binarization. Reference [23] proposes using 0 and 1 to represent the weight and activation value. In this method, they achieve a 32 times reduction in model size and seven times faster on GPU at run-time. Nevertheless, the method of weight quantization depends on specific hardware and the accuracy of the quantized network will decrease a lot, especially in the case of very few clustering categories.

**Knowledge distillation**. Knowledge distillation [24,25] is widely used in model compression and transfer learning. The complex network with strong learning ability distills the “knowledge” represented by the characteristics and transfers it to the network with a small number of parameters and weak learning ability. Ref. [26] explores that knowledge distillation can be integrated into one of the pruning methodologies, namely pruning filters [18], as the compression technique, to enhance the accuracy of the pruned model.

**Efficient architectures**. Another valid direction is to design efficient network architectures. SqueezeNet [7] substitute 1 × 1 filters for 3 × 3 convolutional filters, decrease the number of input channels corresponding to 3 × 3 filters, and downsample late in the network so that convolutional layers have large activation maps. Additionally, they use model compression techniques [16] to compress SqueezeNet to less than 0.5 MB. MobileNet [6] uses depth-wise separable convolutions to structure lightweight DNNs. Through the adoption of depth-wise convolution, the following results can be achieved: (1) Reduce the number of parameters; (2) Improve the operation speed. ShuffleNet [27] shuffles channels orderly to form a new set of feature maps, which solves the problem of “poor information circulation” caused by group convolution. Reference [28] proposes a shallow network training strategy to reduce the network’s parameters and computational complexity, furthermore, it also utilizes *FireModule* and factorization technique to further decrease the parameter and improve the feature extraction capability respectively. These lightweight networks are widely used in real-world applications.

## 3. Pruning Network

**Figure 2:**
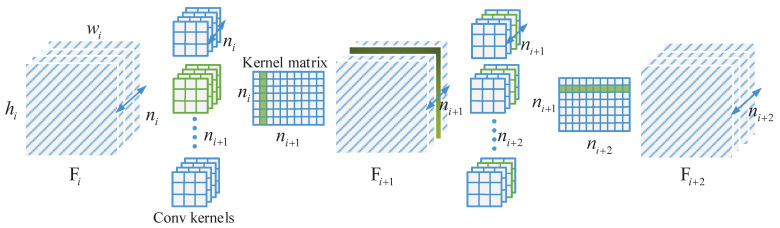
Pruning a channel results in the removal of both the corresponding filter in the previous layer and related convolution kernels in the next layer.

As shown in Figure 2, let $n_{i}$ denote the number of input channels for the *i*th convolutional layer, $h_{i}$ and $w_{i}$ are the height and width of feature maps respectively, and $n_{i+1}$ means the number of total filters for the *i*+1th convolutional layer. The convolutional layer convolves the input feature maps $F_{i}$ through *n*+1 filters to obtain the output feature maps $F_{i+1}$, which are used as input channels for the next convolutional layer. Each 3D filter is composed of *n* 2D kernels of size k×k, and all filters of *i*th convolutional layer constitute a kernel matrix. In the convolution process, *i*+1 filters generate *n*+1 channels, when a channel in *i*+1th convolutional layer is pruned away, a filter in the previous layer will be correspondingly removed and, in addition, the convolution kernel corresponding to the already pruned channel in all filters of the next layer will also be removed. In the case of normal, the number of operations of *i*th convolutional layer is $n_{i}n_{i+1}k^{2}h_{i+1}w_{i+1}$, while a channel is pruned away, $n_{i}k^{2}h_{i+1}w_{i+1}$ and $n_{i+2}k^{2}h_{i+1}w_{i+1}$ operations will be reduced in the *i*th convolutional layer and *i*+1th convolutional layer respectively. If *m* channels are pruned in *i*+1th convolutional layer, it will reduce $2m/n_{i+1}$ of the computation cost totally.

### 3.1. Determining the Sparse Level

In this part, we mainly discuss why we chose the channel pruning method. There are two different pruning methods, one is structured pruning, another is unstructured pruning. Structured pruning usually refers to channel-level, filter-level and layer-level pruning, while unstructured pruning usually prunes weights in the kernel matrix. Unstructured pruning does not remove the unimportant weights, but sets them to zero, so as mentioned above, unstructured pruning relies on specific hardware or software to accelerate the inferencing process and compress the model. Therefore, the structured pruning method is usually chosen to compress the model and do fast inference in engineering. Also, layer-level pruning is significant for deep networks, while for shallow networks, this method may seriously affect the test accuracy. In our experiment, the channel pruning [20] approach is chosen to prune VGG-16.

### 3.2. Determining Which Channel Should Be Pruned

During the training of the neural network, the data distribution of each layer is likely to be changed after matrix multiplication and nonlinear transformation, with the multi-layer operation of DNNs, the data distribution changes more. As a result, more and more data distributed in the region where the derivative value of the activation function is zero so that the weight cannot be updated and the gradient vanishes. Ref. [29] has proposed Batch Normalization (BN), by reducing internal covariate shift, it can not only avoid the disappearance of the gradient, accelerate the convergence speed, but also alleviate the over-fitting phenomenon to a certain extent. Therefore, it is very common to add BN layers to a neural network. The BN algorithm process during the training of the neural network is given below:

- For the fully connected network, the mean value $\mu_{\beta }$ and standard variance $\sigma_{\beta }$ of the output data of neurons in the upper layer are calculated first.
- Normalizing them to obtain the following Formula (1).
  (1) $$\hat{x_{i}}=\frac{x_{i}+\mu_{\beta }}{\sqrt{\sigma_{\beta }^{2}+\epsilon }},$$
  where $x_{i}$ is the output data of the previous layer, and ϵ is a small value added to avoid the denominator being zero.
- Finally, the data obtained through the above normalization processing are reconstructed to get the following Formula (2):
  (2) $$y_{i}=\gamma \hat{x_{i}}+\beta ,$$
  where γ and β are trainable parameters.

For convolutional neural networks, each channel can be regarded as a neuron by using the strategy of weight sharing, so only two parameters γ and β need to be saved for each channel. Ignoring the influence of β, it can be considered that the larger γ is, the larger the output value $y_{i}$ of the reconstructed data will be. Therefore, γ can be chosen as the scaling factor, and sparse regularization be made on it, and then those channels with small scaling factors can be removed to obtain a compact network. The loss function needs to be optimized in the training process is definite by:

(3) $$L=\sum_{\left(x,y\right)}l\left(R\left(x,W\right),y\right)+\lambda \sum_{\gamma \in \Gamma }.\gamma^{2}$$

The above Formula (3) has two parts, the first sum-term corresponds to the normal training loss of a CNN, where *x* and *y* are the input value and real label of the sample in the dataset respectively, W is the weight matrix, and *R* is the activation function. The second sum-term is ridge regression for γ. λ is an adjustable parameter used to balance these two terms. The larger the λ is, the greater the penalty for γ will be and the closer γ will be to zero, which is more conducive to the compression model. In our experiment, λ was set to $10^{-4}$.

**Figure 3:**
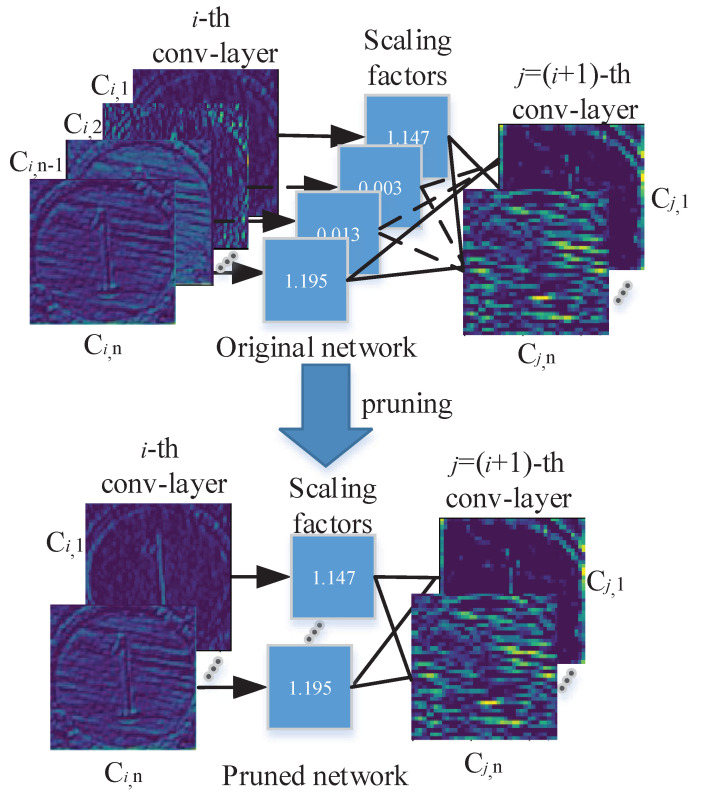
We use the trainable parameter γ in the batch normalization layer as the scaling factor and each channel corresponds to a scaling factor. After sparse these scaling factors, the channels with small scaling factors are taken as trivial channels and removed. After pruning, we can obtain a slim network.

By continuously optimizing the aforementioned loss function, scaling factors of different channels at each layer can be obtained, and then the channel can be pruned with a small scaling factor from a well-trained model for computational efficiency while minimizing the precision drop. In this process, the corresponding filters of the previous layer and kernels of filters for the next layer are also removed (see Figure 3).

### 3.3. Determining the Pruning Ratio and Method

**Figure 4:**
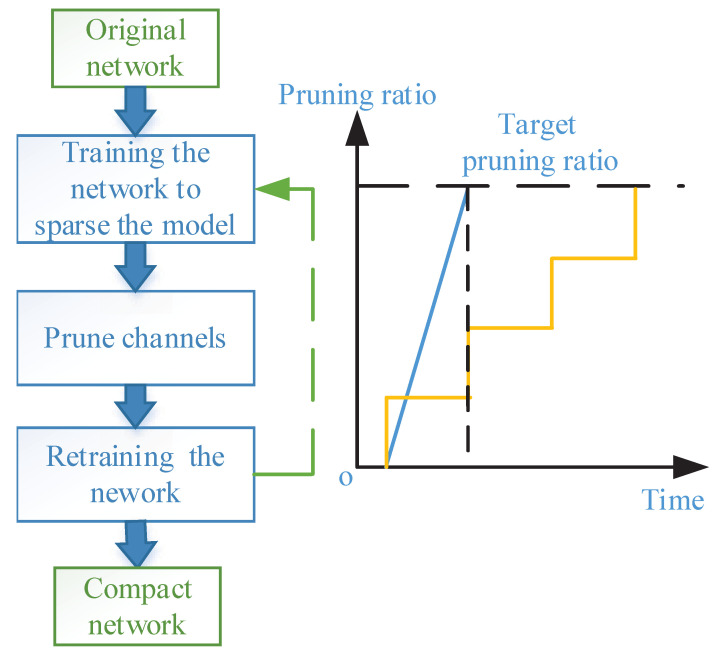
Left: Flowchart of different pruning methods, the dotted-line represents iterative pruning. Right: The blue line represents the One-shot Pruning method and the yellow line represents the Iterative Pruning method.

After conducting L2-norm for γ, most scaling factors are near zero after training the network. To determine the pruning ratio, there are two approaches: (a) A global threshold can be selected, which is determined by the percentage of channels that need pruning in the total number of channels. In the whole network, channels corresponding to scaling factors less than this threshold will be removed; (b) A pruning ratio is set for each layer to determine the threshold. Although the problem of measuring the capacity and redundancy of each layer of neural networks has not been solved, this method is more reasonable than selecting the global threshold, because the redundancy of each convolutional layer is different. If only one global threshold is selected, more channels in the deeper convolutional layer are likely to be pruned due to the deeper layer contains more channels. In our experiment, we empirically determine the number of filters to be pruned for each layer based on their sensitivity to pruning [18]. By removing channels with scaling factors lower than the threshold, a more compact network could be obtained, which has fewer parameters and footprint memory, as well as less computation. Similarly, there are two common strategies for pruning: (a) One-shot Pruning; (b) Iterative Pruning. For a certain target pruning ratio, One-shot Pruning based on the magnitude of scaling factors, prune the channel with a small scaling factor at once and then retrain the network to regain comparable accuracy. Iterative Pruning will be carried out in multiple stages. Firstly, the network will be sparse, part of the channel will be pruned, and then the network will be sparse again, and the cycle will be repeated until the target value is reached (see Figure 4). For deep networks, like resnet50 or resnet110, it can be extremely time-consuming to prune channels and retrain DNNs repeatedly. In this study, the One-shot Pruning method is utilized to prune VGG-16, because it is unacceptable to sacrifice time cost to achieve a higher speedup ratio and compression ratio.

## 4. Experiments

### 4.1. Dataset

**Figure 5:**
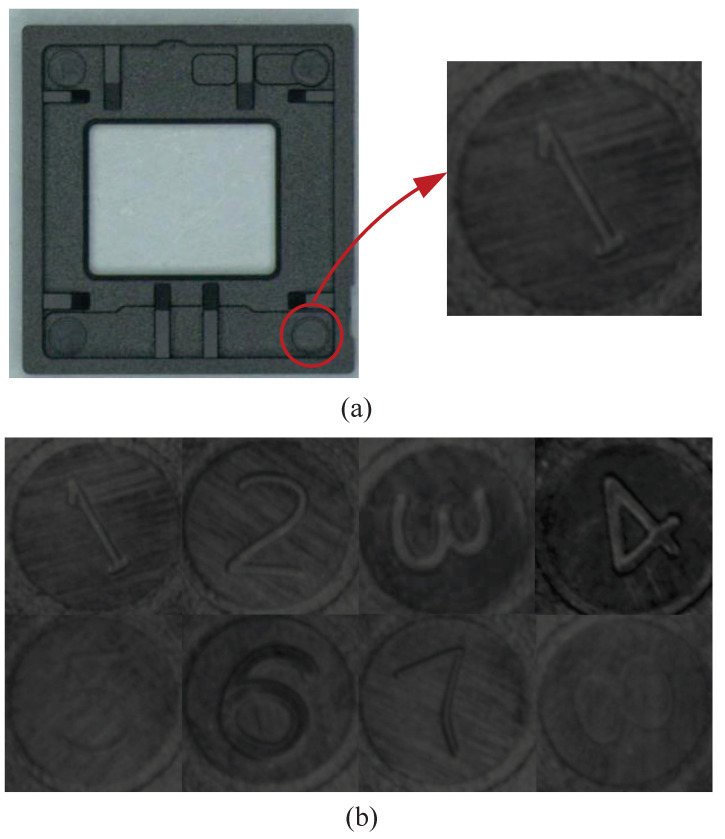
The number label on the workpiece and its location. (**a**) The industrial electronic equipment parts and the captured digital number. (**b**) The Digital number on some industrial electronic equipment parts, from 1 to 8, respectively representing the corresponding mold number of different workpieces.

**LN**. To test and train neural networks, the data collected from the industrial working field is leveraged as a dataset and called LN. As shown in Figure 5, there are number labels on some industrial electronic equipment parts, from 1 to 8, respectively representing the corresponding mold number of different workpieces. In the process of checking the working state of the mold, we can determine whether the workpiece mold is working normally according to the number of times that the number label on some faulty workpiece appears, when the number of times the label on the faulted workpiece appears more, it indicates that the corresponding mold may work abnormally. To obtain the data set, the method of object detection was adopted to locate the workpiece label number and then used image segmentation to capture the label image [30]. A standard data augmentation scheme (rotation, resize, brightness, contrast vary randomly and random injection noise) is adopted. We can obtain a dataset consists of number label images with a resolution 152 × 152. The train and test sets contain 50,000 and 10,000 images respectively.

**CIFAR-10**. In order to test whether the proposed method can get consistent results on other datasets, we conducted relevant experiments on CIFAR-10.CIFAR-10 is drawn from 10 classes. The train and test sets contain 50,000 and 10,000 images respectively. We recorded the best results during the training and fine-tuning process. A standard data augmentation strategy is used, including shifting and mirroring. The input data is normalized using channel means and standard deviations.

**MNIST**. MNIST is a handwritten digit dataset containing 60,000 training images and 10,000 testing images. To compare the performance of LeanNet to the other networks, the experimental result is also recorded.

### 4.2. Network Models

**VGGNet**. VGGNet is a deep convolutional neural network developed by Oxford University’s visual geometry group and researchers at Google Deepmind [5]. VGGNet explores the relationship between the depth of a convolutional neural network and its performance. Multiple types of deep convolutional neural networks (VGG-13, VGG-16, VGG-19) can be obtained by stacking the convolutional layers and the maximum pooling layers. Because of the strong extensibility of VGGNet, and the generalization of migrating to other image data is excellent, it is widely used to extract image features and do image classification tasks. Since the resolution of the image we obtained was 152 × 152, the structure of VGGNet should be adjusted accordingly. To reduce the number of parameters in the fully connected layer, the fully connected layer is replaced with the global average pooling layer [31].

**MobileNet**. MobileNet was proposed by Google in 2016 [6], and its idea is mainly derived from Xception [32]. In order to reduce a large amount of computation with slightly reduced precision, it uses a depthwise separable convolution module, which contains depth-wise and point-wise convolution. The computation required for a normal convolution operation is: $M_{F}\cdot M_{F}\cdot K\cdot N\cdot M_{K}\cdot M_{K}$, where $M_{F}\cdot M_{F}$ and $M_{K}\cdot M_{K}$ means the feature map size and kernel size respectively, *K* means the number of channels and *N* means the number of filters. The computation for depth-wise convolution is: $M_{F}\cdot M_{F}\cdot K\cdot M_{K}\cdot M_{K}$, and for point-wise convolution is: $M_{F}\cdot M_{F}\cdot K\cdot N$. By using depthwise separable convolution module, it get a reduction in computation of:

$$\begin{array}{cc} \; & \frac{M_{F}\cdot M_{F}\cdot K\cdot M_{K}\cdot M_{K}+M_{F}\cdot M_{F}\cdot K\cdot N}{M_{F}\cdot M_{F}\cdot K\cdot N\cdot M_{K}\cdot M_{K}}\\ \; & =\frac{1}{N}+\frac{1}{M_{K}\cdot M_{K}}. \end{array}$$

According to the kernel size, depthwise separable convolution would save about $M_{K}\cdot M_{K}$ times less computation than standard convolutions. MobileNet also has several different types of structures, and in the comparison experiment, MobileNet-V1 is chosen.

**SqueezeNet**. [7] proposes SqueezeNet in 2016. It is composed of several *FireModule*, which consists of the Squeeze module and the Expand module. The squeeze module uses 1 × 1 convolution kernels for feature dimension reduction and Expand module uses the combination of 1 × 1 and 3 × 3 convolution kernels for feature dimension raising. SqueezeNet can significantly reduce network parameters, achieves Alex-level accuracy with 50× fewer parameters. We also compared SqueezNet with LeanNet in our experiments.

### 4.3. Implementation Details

For normal training, we train all three networks normally from scratch as the baseline. We use the Mini-batch Gradient Descent as the optimizer. The batch size of VGGNet, SqueezeNet, and MobileNet is set as 32, 64, and 64 respectively. The initial learning rate is set as 0.1, 0.01, and 0.01 for VGGNet, SqueezeNet, and MobileNet respectively, too small a learning rate will result in the weight not being updated and the loss value will not decrease. The learning rate decreased by 10 times every 50 epochs as epoch increased. In addition, we use exponential moving average to enhance the generalization ability of the model, the weight decay and Nesterov momentum are set by $10^{-4}$ and 0.9 respectively. We train 160 epochs for the three different networks. During the sparsity process of VGGNet, the hyperparameter λ, which balances the loss value and sparsity ratio, is set by $10^{-4}$. All other parameters are kept the same as in normal training. In the pruning process, the method to determine the number of channels to be pruned for each layer proposed by [18] is adopted. After pruning channels, new kernel matrices are copied to each layer of the new model, and a compact network was obtained. We can retrain the network that uses the same optimization setting as in normal training to compensate for the decreased accuracy due to pruning. The code is available at https://github.com/liulonghoi/networkcompression.git, (accessed on 28 April 2021).

### 4.4. Results

**Figure 6:**
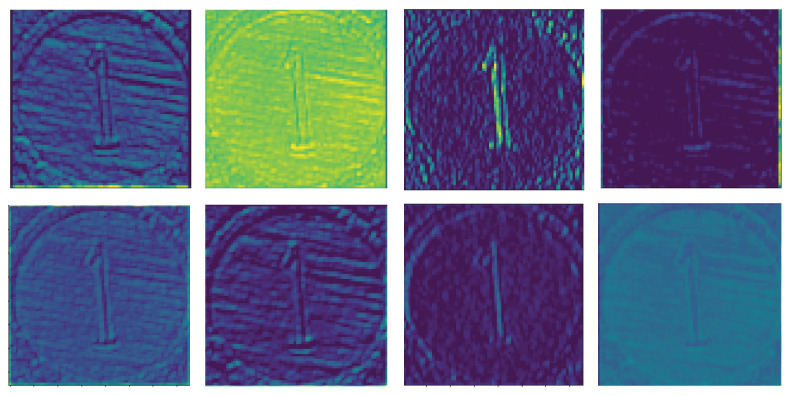
Visualization of 8 channels randomly selected in the first convolutional layer of VGG-16 trained on our dataset. Different channels reflect different information about the input data.

In a convolutional neural network, usually with the increase of network layers, the number of filters contained in the convolutional layer will increase, and the number of channels will also increase, but the size of the feature map will decrease. Different filters represent descriptions of images from different angles and each channel represents different features of the original data. However, some channels do not learn effective feature information during network training, and their values are usually very small, that is, their scaling factors are very small. As shown in Figure 6, eight channels of the first convolutional layer are randomly selected, some channels can reflect the contour, edge and chromatic features of the original image, while some channels are very similar to the input image of the previous layer, indicating that no significant features have been learned. We now evaluate the performance of the proposed LeanNet on our dataset from three aspects.

**Figure 7:**
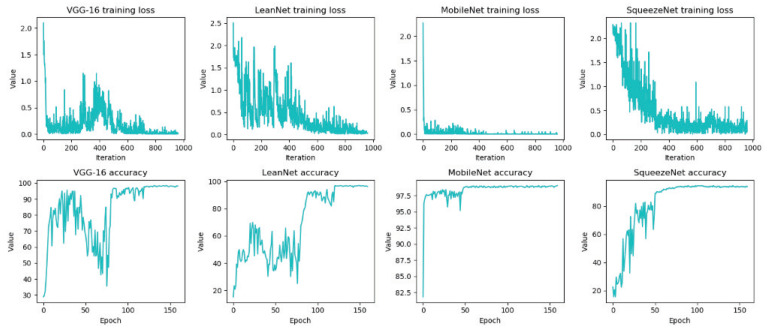
Curves of training loss and test accuracy for different networks.

*Accuracy*. As shown in Figure 7, the accuracy and loss value curves of different network structures in the training process are recorded. The loss value of different network structures oscillate violently at the beginning but eventually converge to the optimal solution. The accuracy of LeanNet is close to the original VGGNet, which proves the redundancy of DNNs. Even if some layers of channels are pruned off by 70%, LeanNet can still achieve the original accuracy through retraining. This process is similar to the biological phenomenon in the mammalian brain, where the number of synapses peaks in early childhood, followed by gradual pruning during its development, but still performances well. As shown in Table 1, in the experiment on the CIFAR-10 dataset, LeanNet reduced the accuracy rate by 1.48% compared to VGG16, but the size of the model was reduced by 26 times. On MNIST dataset, similar conclusions can be drawn that when LeanNet differs from VGG-16 in accuracy by 0.12%, FLOP decreases by 80%, and the real inference time also decreases by nearly 20% under GPU condition and 80% under CPU condition. Judging from the different degrees of decrease in the inference time in the CPU/GPU environment, it can be seen that the important factors affecting the inference speed in the GPU environment are model loading and data IO, rather than the actual recognition consumption of the model.

*Compression ratio*. As shown in Table 1, the number of parameters contained in different networks and the size of model generated under the Pytorch framework are recorded. When 80% channels are pruned, the parameter saving for LeanNet is more than 20×, LeanNet also has an advantage in model size over the other two lightweight networks.

*FLOPs reduction and actual running speed*. Since the computation is mainly concentrated in the convolutional layer, the channel pruning method is effective for increasing the inference speed. As can be seen in Table 1, the inference speed of each model is reported, which is tested by Pytorch. LeanNet has a significant improvement in inference speed on GPU(2080Ti) compared with VGG-16, but will slow down when applied on CPU. Compared to the reduction in FLOPs, MobileNet does not perform well on the GPU for inference speed actually, it may be that cuDNN does not support depth-wise convolution well. SqueezeNet achieves comparable speed-up as LeanNet on GPU with fewer parameters. However, LeanNet costs less inference times under CPU conditions, meanwhile it adopts a special structure that needs to be designed manually. For the network pruning method, it only needs to prune the network required for a specific task, which is relatively flexible.

**Figure 8:**
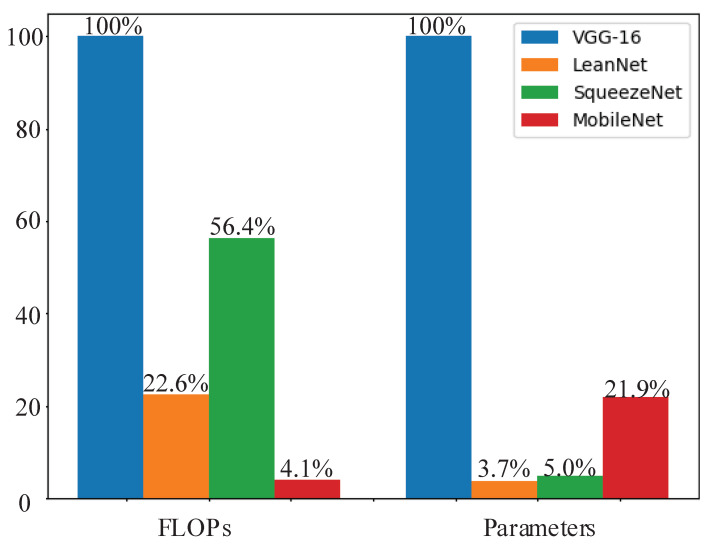
Comparison of parameters and computation under different networks.

To highlight LeanNet’s efficiency, the resource savings for different networks are plotted in Figure 8. Based on the tradeoff among the three aspects of network performance mentioned above, it can be considered that LeanNet achieves the most impressive performance in the actual project.

## 5. Discussion

Considering the influence of hyperparameter λ and the pruning ratio for each layer on the experimental results. In this section, We will analyze their effects in more detail.

**Figure 9:**
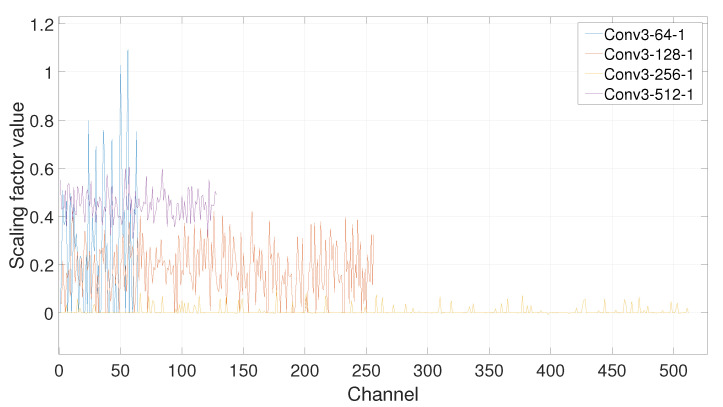
Distributions of scaling factors under different convolutional layers.

**Figure 10:**
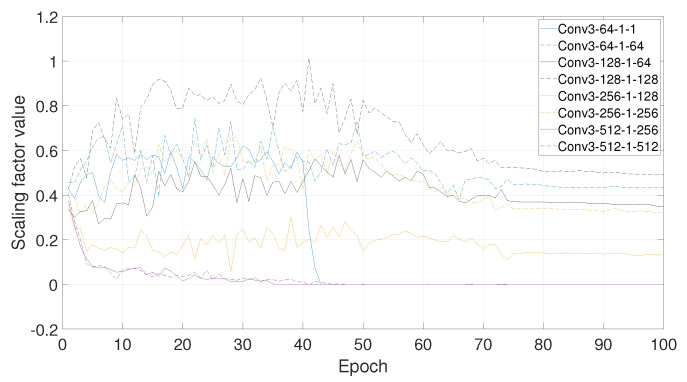
The scaling factor of different channels under different convolutional layers changes dynamically with the increase of the epoch of training rounds. The deeper layer is, the faster the channel’s scaling factor of this layer will to be near zero.

**Effect of Pruning Ratio**. According to [18], for CNNs, such as VGGNet or ResNets, that layers in the same stage(with the same channel size) have a simliar sensitivity to pruning, and moreover, deeper layers are more sensitive to pruning than layers in the earlier stages of the network. Therefore, we can define *p_i_* as the pruning ratio for layers in the *i*th stage, and prune VGG16’s layers with *p_1_* = 40%, *p_2_* = 50%, *p_3_* = 60%, and *p_4_* = 70%. The results are summarized in Figure 9 and Figure 10.

From Figure 9, it can be concluded that after the network training is over, the deeper layer is, the channel’s scaling factor of the layer is smaller. This also proves that the deeper layer is more sensitive to pruning. In this case, if a global pruning rate is set, it will eventually cause more channels in the deeper layer of the network to be pruned, and the deep semantic information extracted by the convolutional neural network will be discarded, which does not meet our expectations. Similarly, it can be seen from Figure 10, in the same layer, the scaling factors of different channels show a gradual decrease as the number of training epochs increases. The scaling factor of some channels will quickly decay to zero, and some others will drop to a certain extent from the initial scaling factor, but will not be forced to be near zero, so they will be retained.

**Figure 11:**
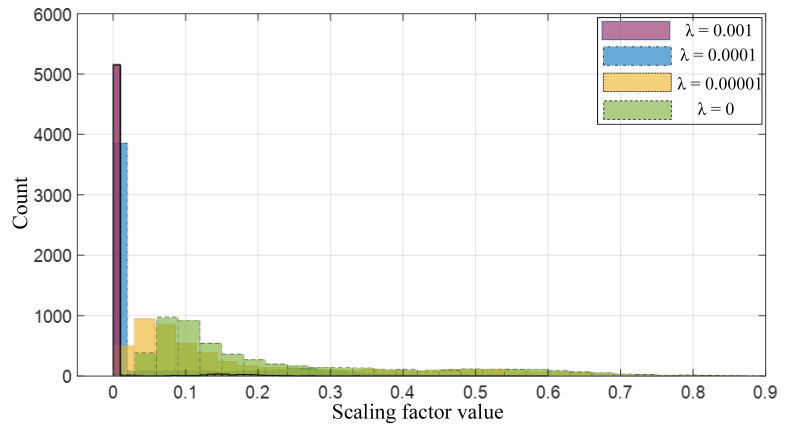
The distribution of scaling factors in four groups of VGG16 under different sparsity regularization (controlled by the parameter λ). As λ increases, the scaling factor becomes sparse.

**Effect of λ**. Considering the influence of hyperparameter λ on network pruning, We further visualize the distribution of different λ values of the scaling factors in the overall network in Figure 11. For this experiment, we use a VGG16 trained on the CIFAR-10 dataset. It can be found that with the increase of λ, more and more scaling factors are concentrated near 0. When λ = 0, that is, there is no sparsity regularization, and the distribution is relatively flat. When λ = 0.001, almost all scaling factors fall into a small area close to 0. The process of sparsity regularization can be regarded as a channel selection happening in a layer of CNNs, that is, the scaling factor of those futile channels is forced to be near zero. Therefore, in the process of network sparsity, λ needs to be set to an appropriate value, otherwise, when λ is too large, under a certain threshold, more channels will inevitably be pruned, which brings some difficulty to the fine-tuning of the network in the following process.

## 6. Conclusions

In this paper, we prune VGG-16 via the channel pruning method, so as to tackle the problem that DNNs are difficult to deploy on some resource-constrained platforms. It directly leverages the scaling factor γ in BN layers as an agent for channel selection, and some negligible channels can be automatically identified in the training process and then pruned. The efficiency of LeanNet is evaluated on our dataset, LeanNet can simultaneously reduce the model size and computation with small overhead, and does not rely on specific hardware or libraries. Compared to other lightweight networks, LeanNet also achieves the most excellent performance in some real-world application scenarios.

Future work will include exploring the influence of the pruning ratio of each layer on the performance of the model, aiming to design the pruning ratio of each layer more rationally. Moreover, the study should consider how to avoid reducing the recognition accuracy while compressing the model.

## Figures and Tables

**Table 1:** Overall result. The best test accuracy for different networks on three datasets is reported. The last column shows inference time which is tested on one GTX 2080Ti GPU or Intel i5-4210u CPU with batch size 32 respectively, the testing sample set contains 10,000 images totally.

Dataset	Model	Accuracy (%)	FLOP (Giga)	Parameters (M)	Model Size (MB)	Inference Time (s)
LN	VGG-16	98.37	7.02	14.72	117.2	7.56/633.85
	LeanNet	97.17	1.59	0.55	4.5	5.36/120.58
	SqueezeNet	94.37	3.96	0.73	5.9	6.19/453.97
	MobileNet	99.05	0.29	3.22	25.9	5.15/112.01
CIFAR-10	VGG-16	93.43	0.31	14.72	117.2	4.75/387.25
	LeanNet	91.95	0.05	0.55	4.5	2.95/65.27
	SqueezeNet	92.97	0.13	0.73	5.9	3.24/238.59
	MobileNet	91.85	0.01	3.22	25.9	2.79/63.34
MNIST	VGG-16	99.65	0.21	14.72	117.2	3.07/264.35
	LeanNet	99.53	0.04	0.55	4.5	2.47/55.27
	SqueezeNet	99.57	0.09	0.73	5.9	2.26/165.65
	MobileNet	99.45	0.01	3.22	25.9	2.68/61.37

## Data Availability

Publicly available datasets were analyzed in this study. The data can be found here: (MNIST) http://yann.lecun.com/exdb/mnist/, (CIFAR-10) https://www.cs.toronto.edu/~kriz/cifar.html.
